# Orbitofrontal cortex grey matter volume is related to children’s depressive symptoms

**DOI:** 10.1016/j.nicl.2020.102395

**Published:** 2020-08-25

**Authors:** Matthew R.J. Vandermeer, Pan Liu, Ola Mohamed Ali, Andrew R. Daoust, Marc F. Joanisse, Deanna M. Barch, Elizabeth P. Hayden

**Affiliations:** a Department of Psychology, The Brain and Mind Institute, Western University, Western Interdisciplinary Research Building, Room 3190, 1151 Richmond St., London, ON N6A 3K7, Canada; b Department of Psychiatry, Washington University School of Medicine, 4444 Forest Park Avenue, Suite 2100, St. Louis, MO, USA; c Department of Psychology, Washington University, St. Louis, MO, USA; d Department of Radiology, Washington University School of Medicine, St. Louis, MO, USA

**Keywords:** Voxel-based morphometry, Depression, Orbitofrontal cortex, Children, Reward

## Abstract

Adults with a history of depression show distinct patterns of grey matter volume (GMV) in frontal cortical (e.g., prefrontal cortex, orbitofrontal cortex) and limbic (e.g., anterior cingulate, amygdala, hippocampus, dorsal striatum) structures, regions relevant to the processing and regulation of reward, which is impaired in the context of depression. However, it is unclear whether these GMV associations with depression precede depressive disorder onset or whether GMV is related to early emerging symptoms or familial depression. To address these questions, we used voxel-based morphometry (VBM) to examine GMV in 85 community-dwelling children (*M* = 11.12 years, *SD* = 0.63 years) screened for current and lifetime depression. Associations between children’s depressive symptoms (self- and mother-report of children’s symptoms), children’s maternal depression history, and GMV were examined. Although maternal depression history was unrelated to children’s GMV, child GMV in the orbitofrontal cortex (OFC) was negatively related to children’s self-reported depressive symptoms, using both *a priori* ROI and whole-brain analyses. Moderated regression analyses indicated that girls’ GMV was negatively related to girls’ depressive symptoms (as indexed by both self- and mother-report of girls’ symptoms), whereas boys’ symptoms were positively related to GMV. Our findings suggest that brain morphology in the OFC, a region with functional roles in processes relevant to depressive symptoms (i.e., reward-based learning and reward processing), is associated with early depressive symptoms prior to the development of clinically significant depression.

## Highlights

• Healthy children’s orbitofrontal grey matter volume related to depressive symptoms.
• Maternal depression history is unrelated to children’s brain structure.
• Sex moderates relationship between orbitofrontal structure and depressive symptoms.
• Girls have negative orbitofrontal grey mater volume-depressive symptom relationship.
• Boys have positive orbitofrontal grey matter volume-depressive symptom relationship.

## 1 Introduction

With a worldwide lifetime and annual prevalence of 14.6% and 5.5% (Bromet et al., 2011), respectively, major depression (Major Depressive Disorder; MDD) is sometimes referred to as the “common cold of mental illness.” However, this analogy belies the profound personal and societal consequences of MDD (Lépine and Briley, 2011). Globally, depression is associated with an array of negative psychosocial outcomes (e.g., academic failure, marital discord and divorce, occupational impairment; Kessler, 2012), and is a leading cause of disability (Vos et al., 2012), suicide (Bostwick and Pankratz, 2000), and increased mortality related to other, co-occurring health conditions (Cuijpers and Smit, 2002). Importantly, it is now clearly established that children can and do experience depression; for example, epidemiological research has found a 12-month prevalence of 2.7% for children 8 to 15 years of age (Merikangas et al., 2010). This is consistent with a *meta*-analysis of global epidemiological studies of children and adolescent mental disorder prevalence, which found a pooled prevalence estimate of 2.6% for depressive disorders (Polanczyk et al., 2015). Finally, even in the absence of frank depressive disorder, subthreshold depressive symptoms are associated with significant functional impairment in both children (Wesselhoeft et al., 2013) and adults (Rodríguez et al., 2012), and are an established marker of youth risk for future depressive disorders (Klein, 2008; Shankman et al., 2009).

The pervasive and negative sequelae associated with depression underscore the importance of identifying vulnerabilities early in development and improving our understanding of the mechanisms through which these vulnerabilities lead to disorder. Identification of early vulnerabilities is essential for intervention efforts, which may be especially beneficial during childhood, given that neural plasticity is relatively high (Nelson, 2000) and there is a broader window of opportunity for prevention (Merry et al., 2012, Stice et al., 2009). Research aimed at identifying which children are most vulnerable, and the mechanisms by which depression develops, may hold the key to mitigating its often-devastating impact.

### 1.1 Depression risk and vulnerability

Family history of depression marks significantly higher risk for the disorder (Levinson, 2006); indeed, having a first-degree relative (e.g., a parent) with a lifetime history of MDD is associated with approximately three-fold increase in risk (Weissman et al., 2006). Maternal history of MDD is a particularly strong risk factor for depression in offspring (Connell and Goodman, 2002, Klein et al., 2005). Given the high heterotypic continuity and shared etiology between depression and anxiety (Cummings et al., 2014, Kendler et al., 2003), and the preponderance of familial anxiety among those with depression (and vice-versa; Lawrence et al., 2019, Micco et al., 2009), familial anxiety also marks offspring depression risk. Collecting information regarding family history of depression permits “high-risk” designs whereby vulnerable children are identified in advance of the typical age of onset for depression. High-risk designs, focused on youth with a family history but no personal history of disorder, enhance the ability to distinguish between causal processes versus concomitant features or consequences of the disorder (Talati et al., 2013); however, the mechanisms and processes through which markers of risk (e.g., family history) eventuate in disorder are unclear, complex, diverse, and probabilistic. Historically, investigators have focused on predicting diagnostic outcomes in high-risk youth (i.e., the presence or absence of MDD); however, like all mental disorders, depression is characterized by *equifinality* or *etiological heterogeneity* (Cicchetti and Rogosch, 1996). Thus, high-risk youth who ultimately develop depression likely do so via a heterogeneous array of mechanisms, such as cognitive, biological, and personality vulnerabilities (Gotlib and Hammen, 2009). These processes are often referred to as *endophenotypes* (i.e., etiologically parsimonious mechanisms thought to mediate the relationship between genotype and complex disorder phenotypes; Gottesman and Gould, 2003, Insel et al., 2010); as normally distributed, dimensional phenomena, these hold relatively greater reliability and statistical power than dichotomous diagnoses (Klein, 2008). For these reasons, developmental psychopathologists have focused on quantitative processes that may account for why some high-risk youth ultimately develop clinically significant disorders. With respect to the current study, brain structure may serve as an endophenotype for depression.

Importantly, in order to be useful as an endophenotypic marker of disease risk, measurement of the marker must be reliable. Indices of brain structure (e.g., structural magnetic resonance imaging [MRI]) have very high reliability (Wonderlick et al., 2009), especially in comparison to task-based functional MRI (fMRI; Elliott et al., 2019). Despite largely focusing on adults, the literature examining brain structure in those with a history of depression provides hypotheses for particular regions that merit study in high-risk youth.

### 1.2 Brain structure as an endophenotype for depression

Depression is characterized by dysfunction in cognitive, emotional, and behavioural processes related to emotion processing and regulation (Joormann and Gotlib, 2010), responses to reward (Henriques and Davidson, 2000), stress reactivity (Burke et al., 2005, Lopez-Duran et al., 2009), and executive functioning (Rogers et al., 2004). Thus, development of neurobiologically informed models of depressive etiology focuses on brain regions underlying normative functioning of these processes (Drevets et al., 2008), including examining structural differences in these regions between patients with depression and healthy, never-depressed controls.

This literature implicates a complex network of cortico-limbic and cortico-striatal structures involved in the regulation and processing of emotions (e.g., amygdala, hippocampus, anterior cingulate cortex [ACC], prefrontal cortex [PFC]; Davidson et al., 2002, Davidson et al., 2009) and reward (e.g., orbitofrontal cortex, medial PFC, and striatum; Drevets, 2007, Drevets et al., 2008, Eshel and Roiser, 2010). This is consistent with prominent neurobiological theories of depression, which posit structural and functional aspects of these regions contribute to maladaptive changes throughout cortico-limbic and cortico-striatal networks, eventuating in depression. Specifically, Mayberg and colleagues developed a cortico-limbic model of depression (e.g., Mayberg, 1997, Mayberg et al., 1999, Seminowicz et al., 2004) where reduced neural top-down regulation of emotion (via fronto-cortical dysregulation) and/or increased bottom-up emotion processing (via limbic dysregulation) result in the cardinal symptoms of depression (i.e., persistent depressed mood and anhedonia; Mayberg, 1997, Mayberg et al., 1999, Seminowicz et al., 2004). Drevets and colleagues (e.g., Drevets, 2007, Drevets et al., 2008, Price and Drevets, 2010) describe similar neural features as the source of multiple classes of depressive phenotype (e.g., low mood, anhedonia), incorporating additional brain structures relevant to dysregulation of both cortico-limbic *and* cortico-striatal networks.

With respect to empirical studies, meta-analyses indicate that, relative to never-depressed individuals, adults with a history of depression have lower grey matter volume (GMV), concentration (GMC), and structural volume in frontal cortical regions, including PFC (Arnone et al., 2012, Arnone et al., 2016, Bora et al., 2012b, Du et al., 2012, Peng et al., 2016, Sacher et al., 2012) and orbitofrontal cortices (OFC; Arnone et al., 2016, Arnone et al., 2012). Additionally, adults with a history of depression show less GMV and lower structural volume in limbic regions such as the ACC (Arnone et al., 2016, Bora et al., 2012a, Bora et al., 2012b, Du et al., 2012), amygdala (Arnone et al., 2016, Bora et al., 2012b, Sacher et al., 2012), and hippocampus (Arnone et al., 2016, Arnone et al., 2012, Bora et al., 2012b, Du et al., 2012), as well as reductions in dorsal striatal (i.e., caudate nucleus and putamen) GMV and structural volumes, relative to never-depressed control subjects (Amico et al., 2011, Arnone et al., 2012). Importantly, these findings are consistent with the aforementioned neurobiological theories of depression (Drevets et al., 2008, Mayberg et al., 1999, Mayberg, 1997, Price and Drevets, 2010, Seminowicz et al., 2004), as do findings that depressive symptoms are negatively associated with GMV in the OFC, PFC, and cingulate (Chen et al., 2007, Vasic et al., 2008).

### 1.3 Brain structure in depression risk

While the structural differences identified in studies of people with depression may be indicative of pre-existing vulnerability, it is also plausible that they are *caused* by the disorder or its treatment (i.e., scar effect). A smaller literature, reviewed below, has explored brain structure in those at risk for the disorder without a personal history of depression.

#### 1.3.1 Familial depression and brain structure

Never-depressed adults with a family history of depression tend to have decreased hippocampal volume (Amico et al., 2011, Baaré et al., 2010, Carballedo et al., 2012, Rao et al., 2010); however, both *increases* (Romanczuk-Seiferth et al., 2014) and *no* differences (Mannie et al., 2014) in hippocampal volume have also been reported. Similarly the amygdala (Munn et al., 2007, Romanczuk-Seiferth et al., 2014, Saleh et al., 2012), dorsolateral PFC (dlPFC; Amico et al., 2011, Carballedo et al., 2012, Romanczuk-Seiferth et al., 2014), and medial PFC (mPFC; Amico et al., 2011, Carballedo et al., 2012, Ozalay et al., 2016) are also inconsistently related to family history in non-depressed adults.

There is a small literature examining brain structure in never-depressed youth with and without a family history of depression. Youth amygdala volume and familial history of depression are inconsistently related, with some studies finding that a family history of MDD is associated with smaller amygdalar volumes (Chai et al., 2015), and others finding no differences (van der Plas et al., 2010). Boys and girls may also differ in brain-risk associations; for example, depressive symptoms predicted boys’ ACC volume but not girls’ in never-depressed youth with a familial history of depression (Boes et al., 2008). While intriguing, these studies are limited by examining youth who vary widely in age; for example, both Boes et al., 2008, van der Plas et al., 2010 included seven- to seventeen-year-olds in their studies. Wide age ranges are problematic for studies of youth, as it is unclear whether structural associations reported in the aforementioned studies are reflective of risk prior to the typical age of onset for depression or are largely driven by structural changes in the brain that occur in adolescence (e.g., reductions in grey matter [GM] and increases in white matter [WM]; Sowell et al., 2002, Spear, 2013). Even when age is covaried in analyses, including children who vary widely in age may render results more challenging to interpret than recruiting children who fall within a narrow age range.

#### 1.3.2 Maternal depression and brain structure

A maternal history of depression is especially strongly linked to depression risk in children and adults (Connell and Goodman, 2002, Klein et al., 2005); thus, other high-risk studies have focused specifically on the relationship between *maternal* depression history and brain structure in never-depressed children. In 55 never-depressed 9- to 15-year-old girls, those with a recurrent maternal history of depression had lower hippocampal GMC and structural volume relative to low-risk children (Chen et al., 2010). Using a region-of-interest approach, maternal history of recurrent depression was associated with thinner cortical GM in bilateral fusiform gyri of never-depressed girls (*N* = 14), compared to girls with no maternal history of mental disorder (*N* = 23; Foland-Ross et al., 2016). Ozalay and colleagues (2016) found that never-depressed daughters of mothers with recurrent depression had significant GMV reductions in the right temporoparietal region, bilateral insula, and right dlPFC, relative to never-depressed daughters of never-depressed mothers. Ozalay et al. (2016) also found maternal history of recurrent depression was associated with increased GMV in the left middle temporal cortex. These studies suggest that a maternal history of depression is correlated with daughters’ brain structure, even in the absence of offspring disorder; however, it is unclear whether these findings generalize to boys as well.

While promising, findings regarding brain structure in high-risk children and adults are mixed, possibly due to several factors. First, rather than directly interviewing family members, investigators oftentimes use participants’ reports of their family members’ psychopathology history, a methodologically limited approach subject to an array of biases (Kendler et al., 1991, Milne et al., 2009). Further, given that recurrent depression is more heritable than single episodes (Fernandez-Pujals et al., 2015), using recurrent depression history as an index of children’s risk may be a more powerful marker of vulnerability. Finally, given that the limited studies available on the relationship between children’s brain structure and maternal depression history have focused exclusively on girls, work including both boys and girls is needed.

#### 1.3.3 Sex differences in depression and brain structure

Depression is approximately twice as prevalent in women compared to men (Nolen-Hoeksema and Hilt, 2009), and being female is a significant prospective predictor of depression (Klein et al., 2013). The reasons for this well-established pattern are complex and heterogeneous, likely involving both biological and psychosocial mechanisms. Sex differences in prevalence suggest the possibility that women and men differ on average in the degree to which vulnerability processes are present; however, it is also possible that women are more impacted by these vulnerabilities, even in the absence of mean differences (i.e., a sex-by-vulnerability interaction). For example, studies of cognitive risk (e.g., Mezulis et al., 2010) show that the longitudinal relationship between stress and depression is stronger for girls than boys, and work from our group (Daoust et al., 2018, Kryski et al., 2013) indicates that stress reactivity is more strongly associated with internalizing symptoms in girls than boys.

Few studies have examined sex differences in the relationship between brain structure and depression; however, brain structure in regions related to emotion/reward processing may be more strongly related to depression risk in girls. For example, Kong et al. (2013) found that reductions in limbic (e.g., bilateral amygdala and hippocampus) GMC were associated with depression in women, while men with depression had reduced GMC among striatal regions (bilateral caudate, left ventral striatum). Similarly, Vulser and colleagues (2015) reported that decreased medial PFC GMV mediated the relationship between subclinical depressive symptoms at 14-years-old and major depressive episodes at age 16 for girls but not boys. These few studies suggest that the relationship between depression risk and brain structure may differ by sex.

In addition to sex-based differences in depression risk and vulnerability, it is important to acknowledge that neurodevelopment is also characterized by sexual dimorphisms. Specifically, females consistently show smaller GM and WM volumes across the brain and developmental stages (Lenroot et al., 2007); however, after controlling for differences in total brain size, females have *proportionately* greater volumes in some anatomical regions (i.e., greater GMV in frontal lobes and greater corpus callosum area; Lenroot et al., 2007). In addition, while both sexes follow an inverted U curve with respect to development of GMV, girls tend to reach peak frontal GMV approximately 1 to 2 years earlier than boys (Lenroot et al., 2007), suggesting that the rate of some aspects of brain development is sexually dimorphic.

### 1.4 The current study

Overall, decreased volume and GMC in a number of frontal cortical (e.g., dlPFC and OFC), limbic (e.g., ACC, amygdala, hippocampus), and striatal structures (e.g., caudate nuclei and putamen) appear to be related to a history of MDD and, with less consistency, to risk for depression among never-depressed individuals, including youth. Importantly, these are regions consistent with prominent cortico-limbic and cortico-striatal theories of depression (e.g., Drevets et al., 2008, Mayberg, 1997, Mayberg et al., 1999, Price and Drevets, 2010, Seminowicz et al., 2004); however, much of this work comes from adults with a history of MDD. Similarly, the less-developed literature investigating the relationship between brain structure and depression risk in never-depressed individuals is also largely based on adults. While important, this work is limited in terms of what it can tell us about brain structure in risk for depression.

In this study, we addressed the limitations of the extant literature in several ways. First, we tested the relationship between depression risk and brain structure in never-depressed children. Additionally, we operationalized risk relatively stringently by only including children of mothers with recurrent depression. Further, we analyzed the relationship between brain structure and both self- and maternally reported children’s depressive symptoms, treating symptoms in the absence of depressive disorder as a marker of risk. A small literature indicates that associations between brain structure and depression differ by sex, although little is known about whether such patterns are related to pre-existing risk versus current depression, and many of the high-risk studies have used all-female samples. We therefore examined whether the relationship between depressive symptoms and brain structure was moderated by sex.

## 2 Material and methods

### 2.1 Participants

Children (*n* = 87) and their mothers were recruited from a larger longitudinal study of children’s depression risk (*N* = 409) that began when children were 3-year-olds. At baseline, children with major medical or psychological problems were excluded, and typical cognitive development was verified using the Peabody Picture Vocabulary Test-Fourth Edition (Dunn and Dunn, 2007). For the current study, children were recruited from the larger longitudinal sample based on maternal history of depression (MH+) drawn from data collected at a previous round of data collection for this study (Liu et al., 2019). Children were considered high-risk based on a maternal history of recurrent major depression (*n* = 26), or a maternal lifetime history of a single major depressive episode *and* a serious anxiety disorder (i.e., any anxiety disorder except a specific phobia; *n* = 3)^1^. Low-risk children had no maternal history of major depression or anxiety disorder (see Procedures and Measures for details). From this sample, 237 families were contacted (58 MH+). Children with any contraindications to the MRI scan (e.g., braces, metallic objects implanted in the body, claustrophobic) were deemed ineligible, leaving a pool of 231 families, from which 110 families agreed to participate (36 MH+). Children from these families were screened as described in the following section to ensure the absence of current or lifetime depressive disorder^2^. Eighty-seven children (29 MH+; 49 boys) participated in the MRI session with 85 contributing usable structural MRI scans (29 MH+; 48 boys).^3^ See Table 1 for demographic statistics of this final sample of 85 children and mothers. These 85 children did not differ from the 25 children who either did not participate in the MRI session or did not contribute useable structural MRI scans, on age, Children’s Depression Inventory, Child Behaviour Checklist-Withdrawn Depressed subscale, Youth Self-Report-Withdrawn Depressed subscale, or Peabody Picture Vocabulary Test (collected at age 3) scores, or frequency distributions of children’s sex or maternal risk status (all *p* > .05).

**Table 1:** Descriptive statistics.

Variable	*M*	*SD*	Frequency
PPVT	112.87	14.16	–
Child Age at MRI Visit	11.12	0.63	–
CBCL Withdrawn/Depressed	1.31	1.79	–
CDI	6.61	5.07	–
YSR Withdrawn/Depressed	3.33	2.71	–
ICV	1616.92	137.46	–
Sex (Male/Female)	–	–	48/37
Risk Group (High/Low)	–	–	56/29

*Note.* PPVT = standardized scores from age 3 Peabody Picture Vocabulary Test; CBCL = Child Behavior Checklist; CDI = Children’s Depression Inventory; YSR = Youth Self-Report; ICV = intracranial volume (cm^3^).

### 2.2 Procedures and measures

Data were collected during four separate assessments of children and their mothers. The first assessment, a phone interview, was conducted with mothers over the telephone and consisted of the parent portion of the Kiddie Schedule for Affective Disorders and Schizophrenia, Present and Lifetime Version (K-SADS-PL; Kaufman et al., 1997) administered by trained graduate students in clinical psychology.

At the second assessment (*M* = 17.86 days, *SD* = 14.51 days after the first assessment), conducted in the participants’ homes, children were administered the K-SADS-PL and completed self-reported symptom and severity measures, including the Children’s Depression Inventory 2nd Edition (CDI^4^; Kovacs, 2011; *α* = 0.83) and the Youth Self-Report (Achenbach and Rescorla, 2001) with the help of trained graduate students in clinical psychology. The K-SADS-PL demonstrated 100% interrater agreement (*N* = 11) for all diagnoses in the current study, including depression^5^. In addition, mothers completed the Child Behavior Checklist (CBCL; Achenbach and Rescorla, 2001); we used the withdrawn-depressed subscale from both the CBCL (CBCL-WD; *α* = 0.72) and the YSR (YSR-WD; *α* = 0.72) as indices of maternally and self-reported child depressive symptoms, respectively (see Supplementary Figs. 1–3 for histograms of symptom distributions).

During the third assessment (*M* = 17.04 days, *SD* = 20.03 days after the second assessment), children participated in a laboratory social stressor task; these data are not used in the current analyses. Mothers were also interviewed during this visit by trained graduate students in clinical psychology to assess lifetime history of psychopathology using the Structured Clinical Interview for the DSM-IV-TR Axis I Disorder Non-Patient Edition (SCID; First et al., 2002). As all mothers had completed a SCID several years prior as part of the larger longitudinal study, we focused solely on the interval since participants’ last SCID. The SCID demonstrated good inter-rater reliability for specific diagnoses and for lifetime history of any depressive episodes (Kappa = 1.00, *N* = 10). Finally, in keeping with best practices for scanning children (de Bie et al., 2010), children completed a “mock scan” session during this visit in a replica MRI system in order to prepare them for the fourth and final visit (MRI visit). During the mock scan, the upcoming MRI session procedures were explained and children were given the opportunity to ask questions. Finally, structural and functional MRI scans were acquired from children during an MRI visit held approximately one week after the laboratory visit (*M* = 8.78 days; *SD* = 7.38 days); only the structural data are reported in the current analyses.

### 2.3 MRI data acquisition

Magnetic resonance images were obtained using a Siemens 3T Tim Trio MRI scanner with a 32-channel head RF coil at Western University’s Centre for Functional and Metabolic Mapping. Children’s heads were immobilized during scanning using foam padding in the RF coil. All children wore foam ear buds to dampen scanner noise. Structural images were acquired with a *T*_1_-weighted 3D magnetization prepared rapid gradient echo (MPRAGE) sequence (1 × 1 × 1 mm voxel size, repetition time (TR) = 2300 ms, echo time (TE) = 2.98 ms, field of view (FOV) = 256 mm), 192 slices.

### 2.4 VBM preprocessing

Initially, all raw DICOM scans were reviewed and converted into NIFTI format, using MRICRON software (Rorden et al., 2007). VBM preprocessing was conducted using default settings for Computational Anatomy Toolbox (CAT12, https://dbm.neuro.uni-jena.de/cat/), an extension of SPM12 (Wellcome Trust Center for Neuroimaging, London, UK), and MATLAB 9.5 (Mathworks, Inc., Natick, MA). *T*_1_-weighted images were bias, noise, and global intensity corrected prior to spatial normalization to the MNI152 template using the DARTEL algorithm (Ashburner, 2007). Next, normalized images were segmented into GM, WM, and cerebrospinal fluid (CSF; Ashburner and Friston, 2005) and written as modulated normalized volumes, allowing for interpretation of localized grey matter volume (GMV). Intracranial volumes (ICV) were calculated during segmentation for use as a nuisance variable during statistical analyses. Quality assurance was conducted via visual inspection and an automated quality check protocol embedded in CAT12, leading to the exclusion of one participant. All scans were then spatially smoothed using a 6 mm (FWHM) Gaussian smoothing kernel and resampled into 1.5 × 1.5 × 1.5 mm voxel size.

### 2.5 Data analyses

SPM12 was used to analyze VBM data. All VBM analyses included age, sex, and intracranial volume (ICV) as covariates. Analysis of covariance (ANCOVA) was conducted to test differences in GMV between high- and low-risk children in both *a priori* regions of interest (ROI) and whole-brain analyses. We also used multiple regression to examine associations between children’s depressive symptoms (i.e., CBCL-WD, CDI, and YSR-WD) and GMV in both *a priori* ROI and whole-brain analyses. In addition, we tested statistical interactions between children’s depressive symptoms and child sex, given evidence of sex-based differences in structural brain correlates of depression and depression risk (e.g., Carlson et al., 2015, Kong et al., 2013, Vulser et al., 2015). Specifically, we hypothesized that structure-symptoms associations would be stronger among girls than boys. Therefore, interaction terms were created by taking the product of standardized values of children’s depressive symptoms (i.e., CBCL-WD, CDI, or YSR-WD) and sex. Moderation analyses included the main effects of sex, depressive symptoms, and MH+/MH- status as covariates in the regression model. Average GMV values were extracted from voxel clusters that were significantly associated with an interaction term using MarsBaR, Version 0.44 (Brett et al., 2002) and plotted using R 3.6.1 (R Core Team, 2019) and the *interactions* (Long, 2019a), *jtools* (Long, 2019b), *ggplot2* (Wickham, 2016), and *emmeans* (Lenth, 2019) packages to interpret the interaction.

*A priori* ROIs, selected based on previous work on the relationship between depression risk and brain structure (e.g., Arnone et al., 2016, Arnone et al., 2012, Bora et al., 2012a, Bora et al., 2012b, Du et al., 2012, Lai, 2013) were the anterior cingulate cortex (ACC), bilateral amygdala, bilateral hippocampus, orbitofrontal cortex (OFC), and the dorsal striatum (caudate and putamen). All ROI analyses were conducted using a single ROI mask combining the aforementioned anatomical ROI defined using the Wake Forest University PickAtlas Toolbox, Version 3.0.5 (Maldjian et al., 2004, Maldjian et al., 2003, Tzourio-Mazoyer et al., 2002). Exploratory whole-brain analyses were also conducted using the same statistical models described above. Both ROI and whole-brain analyses were considered significant at *p_FWE_* < 0.05 (random-field theory family-wise error corrected, as implemented in SPM12).

## 3 Results

### 3.1 Associations among major study variables

See Table 2 for bivariate associations between all major study variables. CBCL-WD, CDI, and YSR-WD scores were all positively correlated with one another and with child risk based on maternal history (dummy coded such that 0 = MH- and 1 = MH + ). MH + children had higher CBCL-WD (*t*(81) = -3.10, *p* = .003) and CDI (*t*(82) = -2.24, *p* = .027) scores compared to MH- children; there were no significant differences for YSR-WD scores. Girls tended to have smaller ICV (Table 2) with *t*-tests also showing that boys had significantly larger ICV than girls (*t*(83) = 7.485, *p* < .001). Neither maternal nor self-reported depressive symptoms were associated with child biological sex.

**Table 2:** Bivariate correlations.

	1.	2.	3.	4.	5.	6.	7.	8.
1. Child Age at MRI Visit	–							
2. CBCL Withdrawn/Depressed	−0.17	–						
3. CDI	−0.18	0.50***	–					
4. YSR Withdrawn/Depressed	−0.20	0.47***	0.65***	–				
5. ICV	−0.11	−0.12	−0.01	−0.01	–			
6. Sex	0.12	0.16	0.06	−0.06	−0.64***	–		
7. Risk Group	−0.14	0.33**	0.24*	0.11	0.04	−0.03	–	
8. PPVT	−0.02	−0.06	−0.12	−0.09	−0.05	−0.06	−0.13	–

*Note*. * = *p* < .05; ** = *p* < .01; *** = *p* < .001; CBCL = Child Behavior Checklist; CDI = Children's Depression Inventory; YSR = Youth Self-Report; ICV = Intracranial Volume (cm^3^); PPVT = standardized scores from age 3 Peabody Picture Vocabulary Test; Dummy coding was used for sex (0 = male, 1 = female) and Risk Group (0 = Low maternal risk group, 1 = High maternal risk group).

### 3.2 VBM analyses

#### 3.2.1 Main effects of risk group and depressive symptoms

There were no significant differences in GMV between high- and low-risk children in ROIs (*p_FWE_* > 0.05) or whole-brain analyses (*p_FWE_* > 0.05) using ANCOVA. Additionally, maternally reported children’s depressive symptoms (i.e., CBCL-WD scores) were not significantly related to GMV in any of the ROI-based or whole-brain analyses.

**Fig. 1:**
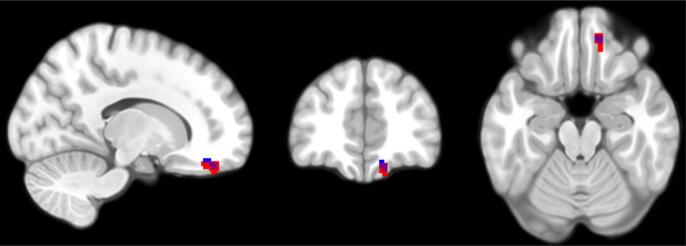
Children's subthreshold depressive symptoms (CDI) are negatively associated with GMV during both ROI regression analysis (clusters highlighted in red for voxels where *p_FWE_* < 0.05) and whole-brain regression analysis (highlighted in blue for voxels where *p_FWE_* < 0.05) regression analyses. (For interpretation of the references to color in this figure legend, the reader is referred to the web version of this article.)

**Fig. 2:**
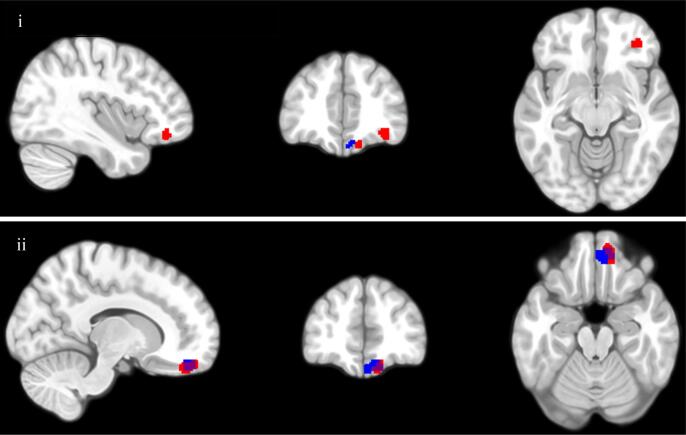
Children’s subthreshold depressive symptoms (YSR-WD) are negatively associated with GMV during both ROI regression analysis (clusters highlighted in red for voxels where p_FWE_ < 0.05) and whole-brain regression analysis (clusters highlighted in blue for voxels where p_FWE_ < 0.05); i = view of lateral OFC cluster; ii = view of medial OFC cluster. (For interpretation of the references to color in this figure legend, the reader is referred to the web version of this article.)

Based on ROI regression analyses of the OFC (Table 3; Fig. 1), children’s self-reported depressive symptoms on the CDI were significantly negatively associated with GMV in a single cluster of voxels in the medial OFC. Similarly, GMV of two independent voxel clusters, the medial and right lateral OFC, was negatively associated with children’s self-reported depressive symptoms on the YSR-WD (Table 4; Fig. 2). The medial clusters identified in regressions using both the CDI and YSR-WD largely overlapped with one another. Depressive symptoms were not significantly related to GMV in any of the other ROI analyses. Exploratory whole-brain voxel-wise analyses identified similarly located clusters of voxels in the OFC where higher CDI (Table 3; Fig. 1) and YSR-WD (Table 4; Fig. 2) were both related to lower GMV (*p_FWE_* < 0.05).

**Table 3:** Regression analyses of grey matter volume for CDI.

Regressor	Cluster Size (mm^3^)	MNI Coordinates (peak voxel)			*p*-value	*Z*
		x	y	z	(FWE corrected)	(peak voxel)
*ROI Analyses*						
CDI × Sex	25.5	−9	62	−8	0.012	4.67
CDI	90	14	45	−21	0.002	4.97

*Whole Brain Analyses*						
CDI	22.5	14	45	−21	0.009	4.97

*Note*. All analyses covaried for the main effects of age, sex, maternal risk group, and total intracranial volume. All *p* values were FWE corrected and refer to cluster level significance. All regression analyses were two-tailed. CDI = Children’s Depression Inventory.

**Table 4:** Regression analyses of grey matter volume for YSR-WD.

Regressor	Cluster Size (mm^3^)	MNI Coordinates (peak voxel)			*p*-value	*Z*
		x	y	z	(FWE corrected)	(peak voxel)
*ROI Analyses*						
YSR-WD	183	12	48	−21	<0.001	5.46
	72	38	42	−11	0.002	4.78

*Whole Brain Analyses*						
YSR-WD	288	11	50	−18	<0.001	5.63
	1.5	39	42	−11	0.037	4.82

*Note*. All analyses covaried for the main effects of age, sex, maternal risk group, and total intracranial volume. All *p* values were FWE corrected and refer to cluster level significance. All regression analyses were two-tailed. YSR-WD = Youth Self-Report Withdrawn/Depressed subscale.

#### 3.2.2 Interactions between child sex and subthreshold depressive symptoms

**Fig. 3:**
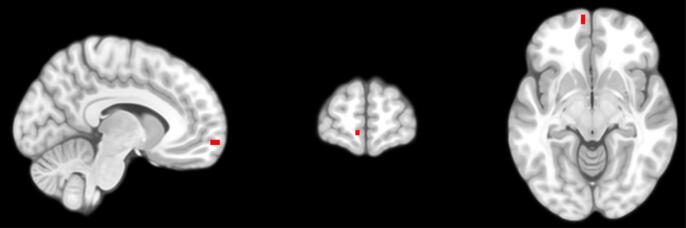
The association between children’s subthreshold depressive symptoms (CDI) and OFC GMV is moderated by sex during ROI analysis (clusters highlighted in red for voxels where *p_FWE_* < 0.05). (For interpretation of the references to color in this figure legend, the reader is referred to the web version of this article.)

**Fig. 4:**
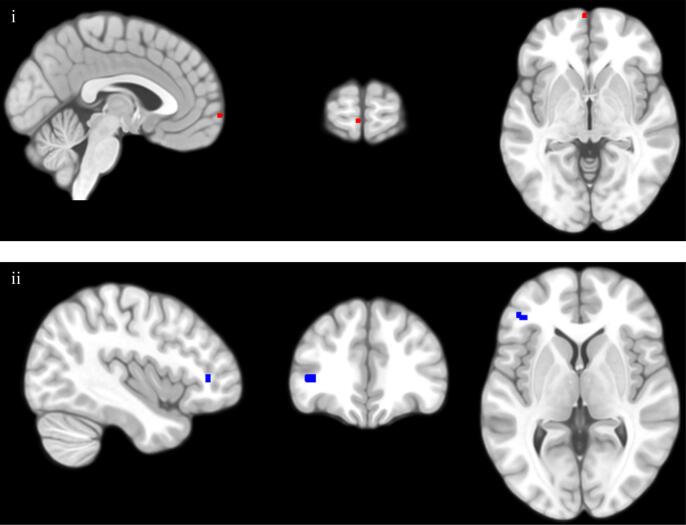
The association between maternal-report of children’s subthreshold depressive symptoms (CBCL-WD) and GMV is moderated by sex both during ROI regression analysis (clusters highlighted in red for voxels where *p_FWE_* < 0.05) and whole-brain regression analysis (clusters highlighted in blue for voxels where *p_FWE_* < 0.05); i = view of medial OFC cluster; ii = view of inferior frontal gyrus cluster. (For interpretation of the references to color in this figure legend, the reader is referred to the web version of this article.)

**Fig. 5:**
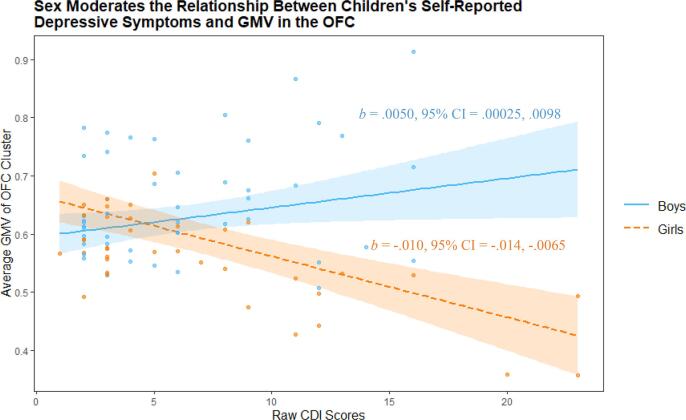
Children’s sex moderates the relationship between self-reported depressive symptoms (according to the CDI) and GMV in an OFC cluster (peak voxel −9, 62, −8), during ROI analysis of the OFC. Highlighted regions indicate 95% confidence intervals.

**Fig. 6:**
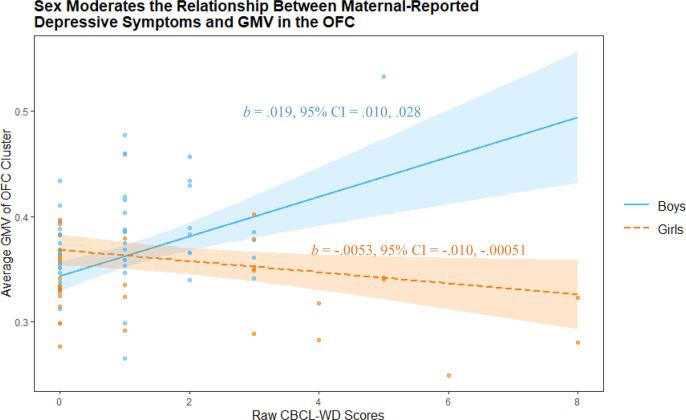
Children’s sex moderates the relationship between maternal report of children’s depressive symptoms (according to the CBCL-WD) and GMV in an OFC cluster (peak voxel −3, 68, −3), during ROI analysis of the OFC. Highlighted regions indicate 95% confidence intervals.

The relationship between CDI and GMV during *a priori* ROI analysis of the OFC was significantly moderated by the sex of child participants (Table 3; Supplementary Table 1; Fig. 3). A similar effect was found whereby the relationship between CBCL-WD and GMV in the OFC ROI were also moderated by sex (Table 5; Supplementary Table 2; Fig. 4). In both cases, simple slopes analyses using the mean GMV of respective significant voxel clusters indicated a significant relationship between GMV and both boys’ and girls’ depressive symptoms (indexed via the CDI and CBCL-WD), although the association was negative for girls and positive for boys (Fig. 5, Fig. 6). Sex did not significantly moderate the relationship between YSR-WD and GMV. Additionally, no significant interactions were identified in any of the other ROI analyses.

**Table 5:** Regression analyses of grey matter volume for CBCL-WD.

Regressor	Cluster Size (mm^3^)	MNI Coordinates (peak voxel)			*p*-value	*Z*
		x	y	z	(FWE corrected)	(peak voxel)
*ROI Analyses*						
CBCL-WD × Sex	6	−3	68	−3	0.029	4.38

*Whole Brain Analyses*						
CBCL-WD × Sex	27	−41	38	6	0.007	5.34

*Note*. All analyses covaried for the main effects of age, sex, maternal risk group, and total intracranial volume. All *p* values were FWE corrected and refer to cluster level significance. All regression analyses were two-tailed. CBCL-WD = Child Behavior Checklist Withdrawn/Depressed subscale.

**Fig. 7:**
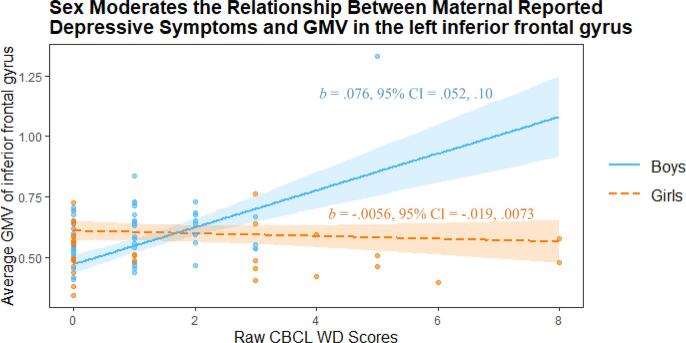
Children’s sex moderates the relationship between maternal report of children’s depressive symptoms (according to the CBCL-WD) and GMV in a cluster (peak voxel −41, 38, 3) in the left inferior frontal gyrus, during whole brain analysis. Highlighted regions indicate 95% confidence intervals.

Exploratory whole-brain analysis identified a cluster in the left inferior frontal gyrus where the relationship between GMV and depressive symptoms (indexed via maternal-reported CBCL-WD) was significantly moderated by children’s sex (Table 5; Supplementary Table 3). Simple slopes analysis indicated that boys’ GMV and maternally reported symptoms (i.e., CBCL-WD) were significantly positively related, while the relationship was non-significant among girls (Fig. 7).

## 4 Discussion

We investigated the relationship between brain structure and an established marker of children’s depression risk, namely a maternal history of recurrent depression (or depression and serious anxiety disorder). Contrary to our expectations, children at high- and low-risk for depression, according to maternal history, did not differ in GMV. This was especially surprising given that children’s maternal risk was relatively stringently defined relative to other studies, and our comparison group of children was drawn from mothers without any history of depression or anxiety. However, while children of mothers with a history of recurrent depression are at relatively higher risk for developing the disorder themselves (Connell and Goodman, 2002, Klein et al., 2005), not all offspring of depressed mothers become depressed, and some develop other forms of psychopathology. Thus, not all children with a maternal history of depression inherit risk, including what may be relatively specific risk marked by brain structure. It is also possible that our high-risk children have risk mechanisms other than those captured by brain structure. Finally, aspects of brain structure that distinguish children with and without a maternal depression history may emerge later in development, a possibility worth exploring in other studies as well as in follow-up assessments of this sample.

In analyses of brain structure-symptom associations, we found that GMV in the medial OFC was significantly negatively associated with children’s self-reported subthreshold depressive symptoms (i.e., CDI and YSR-WD) using both *a priori* ROI and exploratory whole-brain analyses. Furthermore, children’s self-reported depressive symptoms (i.e., YSR-WD) were also negatively associated with GMV in the right lateral OFC, exclusively at the ROI level of analysis. These associations were found solely with children’s self-reported symptoms based on the YSR-WD, and not the CDI nor the CBCL-WD scale (maternal report), perhaps capturing some aspect specific to social withdrawal being more specifically measured using the self-reported YSR-WD. Given the known homotypic continuity of early depressive symptoms with later depressive disorder (Cuijpers and Smit, 2004, Klein et al., 2013, Shankman et al., 2009), these findings highlight structural brain markers of youth at risk for depression.

Contrary to our expectations, maternal history of depression was unrelated to children’s brain structure in the current study, with all associations with brain structure limited to children’s depressive symptoms. Early subthreshold depression symptoms portend later clinically significant disorder (Cuijpers and Smit, 2004, Klein et al., 2013, Shankman et al., 2009) and can therefore be conceptualized as an index of children’s risk for later disorder; however, we acknowledge that it is more complicated to differentiate between subthreshold symptoms and the disorder itself in terms of understanding causal processes. Having said that, many other indices of putative depression risk (e.g., cognitive styles, Alloy et al., 2000) show conceptual overlap with depression, so this conceptual issue is not limited to the current findings. Given that brain structure and symptoms were related in children rigorously screened for a personal history of depression, our findings speak to brain structure-risk associations that cannot be attributed to a depression history or treatment.

Despite the lack of associations between maternal depression history and children’s brain structure, children’s self- and mother-reported depressive symptoms were significantly associated with *both* brain structure and maternal history of depression (i.e., MH+ and MH− groups significantly differed in CDI and CBCL-WD scores). While children with a maternal depression history are unquestionably at greater risk than children of mothers without depression, this risk is probabilistic rather than deterministic. More specifically, depression is etiologically complex with multiple contributing factors that interact with each other and with the environment. In our sample, elevated symptoms in youth with a maternal history of depression stem from an array of risks that are somewhat distinct from the risk marked by a maternal depression history. Similarly, even though we anticipated group differences in brain structure related to maternal depression, we did not expect to find strong associations. This is consistent with relatively modest estimates of the heritability of depression (Fernandez-Pujals et al., 2015). Integrating other etiologically relevant variables (e.g., cognitive style, environmental stressors, other biological factors) with brain data is an important future direction in mapping youth risk more comprehensively.

Our finding that OFC GMV was negatively related to depressive symptoms in never-depressed children is consistent with the literature on adults with a lifetime history of depression (Arnone et al., 2016, Arnone et al., 2012). The OFC is consistently associated with depression, with meta-analysis showing that a lifetime history of MDD is correlated with a significant decrease in both OFC volume and GMV (Arnone et al., 2016, Arnone et al., 2012). Additionally, lesions in the OFC are associated with depression in adults (MacFall et al., 2001). That said, the bulk of the aforementioned work has been conducted in adults with either current or lifetime history of MDD. Of studies focusing on children at high risk for depression, Chen and colleagues’ (2010) study of brain structure in 12-year-old girls also found no significant risk-GMV association during whole-brain analysis; however, they did report significantly lower GMV in bilateral hippocampi during ROI analyses. That said, Chen et al. (2010) used an uncorrected *p* value during ROI analyses, increasing the chance of false-positive findings. While the current data do not allow for determination of causality, our more stringent analyses indicate morphological features of the OFC (i.e., lower GMV in youth without a history of depression) are related to early vulnerability to depression.

Many depressive symptoms reflect behavior guided by neurofunctional circuits involving the OFC (Drevets, 2007). Perhaps most importantly, the OFC is, both individually and as part of a larger network of structures, involved in the processing of reward and reward-based learning (Delgado et al., 2005, Fettes et al., 2017, Liu et al., 2011, Rolls, 2017). The OFC is thought to be involved in the cognitive encoding of representations of reward outcomes (Klein-Flügge et al., 2013) and tracking the relative value of rewarding stimuli (O’Doherty, 2004). Relatedly, signal detection theory shows that depressive symptoms, especially anhedonia (i.e., deficits in motivation, anticipatory and consummatory pleasure, and reward learning), are associated with reduced reward learning (Kunisato et al., 2012, Pizzagalli et al., 2008, Pizzagalli et al., 2005, Vrieze et al., 2013) that persists even after remission of MDD (Pechtel et al., 2013). Given that anhedonia is a core symptom of depression, characterizing neural structures related to reward processing and reward-based learning in depression risk is an important aspect of understanding the disorder. Our findings that children’s depressive symptoms are related to OFC GMV are consistent with findings of reduced functional activity in the OFC of both adults (e.g., Macoveanu et al., 2014, McCabe et al., 2009, Osuch et al., 2009, Redlich et al., 2015) and children (e.g., McCabe et al., 2012) at risk for, or with a history of, depression, during reward-based tasks.

While the OFC in general is thought to be important for reward processing and reward-based learning, medial and lateral OFC are thought to serve slightly different roles regarding these processes (Elliott et al., 2000, Fettes et al., 2017). Regarding reward-based learning, the medial OFC is putatively responsible for encoding the subjective value of rewarding stimuli and for learning based on probability-based behavioural feedback (Fettes et al., 2017, Kringelbach and Rolls, 2004, Kringelbach, 2005), while the lateral OFC is thought to be involved with reversal learning (e.g., suppressing previously rewarded behavior in favor of new behaviors that were previously unrewarded; Clark et al., 2004, Fellows, 2007, Fettes et al., 2017). Thus, our findings of associations between medial and lateral OFC morphology (regions involved with reward processing; Fettes et al., 2017) with depressive symptoms in never-depressed children are consistent with theories of depression that emphasize maladaptive reward responding as an etiological factor in the disorder (Davidson et al., 2002, Treadway and Zald, 2011). However, we did not investigate functional brain activity during reward processing activities in the current study. Although we have identified structural associations with depressive symptoms in anatomical regions of the brain thought to be associated with reward processes, functional brain studies of non-depressed youth in the context of reward processing are necessary to specifically elucidate this relationship.

In addition to the aforementioned main effects relating brain structure and children’s symptoms, we also tested whether boys and girls differed in the relationship between structure and depressive symptoms, in light of evidence that girls may be impacted more strongly than boys by other putative depression vulnerabilities (Hankin and Abramson, 2001, Mackrell et al., 2013). Depressive symptoms and child sex interacted such that depressive symptoms and OFC GMV were significantly positively related in boys, but negatively related in girls. Our sample size was relatively small for testing interactions, and these effects require replication in other samples; however, the fact that sex similarly moderated both maternally and self-reported depressive symptoms and their relationship to OFC GMV, despite the low intercorrelation between the two measures, suggests that this finding may be robust. The negative association between OFC GMV and depressive symptoms among girls is consistent with previous work focusing on adults with a depression history (Arnone et al., 2016, Arnone et al., 2012); however, the positive association between depressive symptoms and GMV in boys was unexpected. This positive slope may reflect differences in the way that depression presents across sex. For example, epidemiological study has shown that females are significantly more likely than males to experience anhedonic symptoms during depressive episodes (Romans et al., 2007). Further, the positive relationship between OFC GMV and depressive symptoms among boys may be related to the typical pattern of externalizing and reward-focused comorbidities seen among males with depression (i.e., higher rates of comorbid substance use disorders in males, relative to females; Marcus et al., 2005). Of course, these explanations are largely speculative at this point, and further research is needed to adequately explain this pattern of results. Nevertheless, biological abnormalities in the GMV of structures responsible for reward processing and rewarding learning may contribute to an increased vulnerability for depression among girls, but not boys. Similarly, it is also possible that GMV in OFC regions may have opposite relationships with depression risk (i.e., depressive symptoms) among boys and girls, such that greater GMV is a risk factor for boys, whereas decreased GMV is relevant to girls’ risk.

As with other VBM-based studies, the relationship between individual differences in GMV and individual differences in brain *function* remains unclear. To the best of our knowledge, there is no research available directly relating GMV to brain function. Instead, studies linking anatomical and functional differences in the brain typically focus on relating functional connectivity in the brain with structural connectivity (i.e., using white matter tractography; de Kwaasteniet et al., 2013, Nixon et al., 2014). While we have characterized statistical relationships between GMV and depression risk (i.e., subthreshold depressive symptoms) in our sample according to the typical functional role of the identified structural regions, it is possible that these associations do not confer differences in brain function. Future studies are needed to explicitly test the relationship between VBM-based study of brain structure and related differences in brain function in structural regions.

### 4.1 Strengths

Our study has a number of important strengths. We studied children without a personal history of depression, based on rigorous screening procedures, prior to the typical age of onset for depression. This indicates that the structural associations with depressive symptoms that we found are not a consequence of clinically significant depression or its treatment. Our sample was relatively large for neuroimaging studies of high-risk youth. Further, using a community-based sample of mothers and their children, rather than a clinical sample, may increase the generalizability of our findings.

### 4.2 Limitations & future directions

Despite the strengths of our study, results should be considered alongside a number of limitations. First, although up to 50% of all adults will meet criteria for a mental disorder during their lives (Kessler et al., 2005, Kessler et al., 2007), we used strict selection criteria for our “low-risk” group, only recruiting children whose mothers had no history of *any* disorder to this group. This may have limited low-risk children to offspring of especially resilient or healthy mothers, potentially limiting the generalisability of our results. Second, symptom/diagnostic data collection occurred an average of one month prior to MRI acquisition. Given the high stability of depressive symptoms in children and adolescents (Tram and Cole, 2006) and brain structure (Focke et al., 2011) over similar durations, it is unlikely this lag influenced our results. Indeed, treating time between assessments as a covariate did not significantly change our results.

Additionally, although the data used in this study were gathered as part of an ongoing longitudinal study of childhood development, the structural MRI data collected here is the first assessment of brain structure we have for these children. With these cross-sectional data we cannot claim causal relationships between brain structure and depression; however, we plan to continue assessing brain development and psychopathology at subsequent follow-ups, thereby permitting testing of stronger claims about causal mechanisms in the brain-depression relationship. Finally, while we aimed to characterize structural features of the brain as they relate to depression risk *before* onset of depressive disorder or the typical age of onset, the brains of our participants have already undergone considerable maturation from a neurodevelopmental perspective. Future investigations should consider applying similar methodology to samples of even younger children to better characterize the brain-depression risk relationship across early development.

Another limitation concerns other relevant variables not included in the current study. While age was included as a covariate in all analyses of imaging data, participants’ pubertal development was not assessed as part of the current study. Given the age of our sample and the established relationship between pubertal development and the development of depression (Angold and Costello, 2006, Angold et al., 1998), covarying for pubertal development in future studies is an important next step in better understanding these relationships. Finally, human development (including development of the brain and mental disorders) does not exist in a vacuum; the relationship between brain structure and depression risk is most likely influenced by gene-environment interactions and epigenetic changes (Meaney, 2010). Future research should collect more data regarding potentially relevant environmental factors (e.g., adverse childhood events, early parenting behaviour, children’s chronic life stress, etc.) and investigate both the direct effect of environmental variables, beyond maternal depression, as well as their interaction with biology (i.e., genotype, brain-based endophenotypes, sex, stress reactivity, etc.).

## 5 Conclusion

Our results demonstrate that depressive symptoms are associated with brain structure among never-depressed children, specifically in the medial and right lateral OFC. These regions are largely associated with functional roles involving reward processing and reward learning, both functions which are highly relevant to core symptoms of depression (i.e., anhedonia). Reduced GMV in these regions may reflect a pre-existing biomarker for depression, potentially contributing to risk for developing depressive disorders.

## Funding

This work was supported by the Canadian Institutes of Health Research (Grant No. CIHR MOP86458 [to EPH]); the Ontario Mental Health Foundation; Canada First Research Excellence Fund for BrainsCAN; 10.13039/100009408Brain Canada Foundation; the Canadian Institutes of Health Research Frederick Banting and Charles Best Canada Graduate Scholarship Doctoral Award (to MRJV); and Children’s Health Research Institute Quality of Life Initiative.

## Ethics

The study was approved by the Western University Health Science Research Ethics Board. All adult participants signed an informed consent form for themselves and their child prior to participation in this study. Further, all child participants signed an informed assent form for themselves prior to participation in the study.

## CRediT authorship contribution statement

**Matthew R.J. Vandermeer:** Conceptualization, Methodology, Formal analysis, Investigation, Data curation, Writing - original draft, Writing - review & editing, Visualization, Funding acquisition. **Pan Liu:** Data curation, Writing - review & editing. **Ola Mohamed Ali:** Investigation, Data curation, Writing - review & editing. **Andrew R. Daoust:** Investigation, Data curation, Writing - review & editing. **Marc F. Joanisse:** Supervision, Writing - review & editing, Funding acquisition. **Deanna M. Barch:** Supervision, Writing - review & editing, Funding acquisition. **Elizabeth P. Hayden:** Supervision, Conceptualization, Methodology, Writing - review & editing, Funding acquisition.

## Declaration of Competing Interest

The authors declare that they have no known competing financial interests or personal relationships that could have appeared to influence the work reported in this paper.

## Footnotes

1 We excluded specific phobia and social anxiety limited to public speaking given that these are less heritable, less impairing, and potentially weaker markers of children’s internalizing risk (Kendler, Neale, Kessler, Heath, & Eaves, 1992)

2 no child was excluded based on current or lifetime depressive disorder

3 One child had orthodontic braces installed between the laboratory visit and the MRI scan; another child had low-quality T1 images despite several attempts at scanning.

4 Despite not meeting criteria for MDD based on the K-SADS-PL, three child participants (*N_MH+_* = 2) had CDI scores>19, which is above the cut-off suggestive of clinically significant symptoms in a community sample (Kovacs, 2011). Excluding these participants from subsequent analyses did not change the pattern of results found using the full sample, although findings were no longer significant after correcting for multiple comparisons and given the reduced sample size.

5 For some K-SADS and SCID diagnoses (e.g., K-SADS depression), no participant met criteria for the disorder. While interviewer agreement on the absence of the diagnosis was 100%, given no variability, we could not compute Cohen’s Kappa in these cases.
