# Supplementary material for: Orbitofrontal cortex grey matter volume is related to children’s depressive symptoms
Source: Neuroimage Clin. 2020 Aug 25;28:102395. doi: 10.1016/j.nicl.2020.102395 (PMC7479290; doi:10.1016/j.nicl.2020.102395)
Supplement: Supplementary data 1 [file mmc1.docx]

| **Supplementary Table 1**  *Moderated regression analysis of grey matter volume for 25.5 mm^3^ cluster with peak voxel at (-9, 62, -8) based on ROI analysis.* | | | | | |
| --- | --- | --- | --- | --- | --- |
| Predictor | *b* | *b*  95% CI  [LL, UL] | *sr^2^* | *sr^2^*  95% CI  [LL, UL] | Fit |
| (Intercept) | .33 | [-0.05, 0.70] |  |  |  |
| ICV | .00** | [0.00, 0.00] | .13 | [.03, .24] |  |
| Sex | .07* | [0.01, 0.13] | .04 | [-.02, .09] |  |
| Age | -.03* | [-0.05, -0.00] | .03 | [-.02, .07] |  |
| Risk | .00 | [-0.03, 0.04] | .00 | [-.00, .00] |  |
| CDI | .01* | [0.00, 0.01] | .02 | [-.02, .07] |  |
| CDIxSex | -.02** | [-0.02, -0.01] | .15 | [.04, .26] |  |
|  |  |  |  |  | *R^2^*  = .564** |
|  |  |  |  |  | 95% CI[.38,.64] |
|  |  |  |  |  |  |
| *Note.* A significant *b*-weight indicates the semi-partial correlation is also significant. *b* represents unstandardized regression weights. *sr^2^* represents the semi-partial correlation squared. *LL* and *UL* indicate the lower and upper limits of a confidence interval, respectively. * indicates p < .05. ** indicates p < .01. ICV = intracranial volume (mm^3^); CDI = Children’s Depression Inventory. | | | | | |

| **Supplementary Table 2**  *Moderated regression analysis of grey matter volume for 6 mm^3^ cluster with peak voxel at (-3, 68, -3) based on ROI analysis.* | | | | | |
| --- | --- | --- | --- | --- | --- |
| Predictor | *b* | *b*  95% CI  [LL, UL] | *sr^2^* | *sr^2^*  95% CI  [LL, UL] | Fit |
| (Intercept) | 0.08 | [-0.10, 0.26] |  |  |  |
| ICV | 0.00** | [0.00, 0.00] | .26 | [.12, .40] |  |
| Sex | 0.02* | [0.00, 0.05] | .03 | [-.02, .07] |  |
| Age | -0.01 | [-0.02, 0.00] | .01 | [-.02, .04] |  |
| Maternal Risk | -0.01 | [-0.03, 0.00] | .02 | [-.02, .05] |  |
| CBCL-WD | 0.02** | [0.01, 0.03] | .10 | [.01, .18] |  |
| CBCL-WDxSex | -0.02** | [-0.03, -0.01] | .13 | [.03, .23] |  |
|  |  |  |  |  | *R^2^*  = .594** |
|  |  |  |  |  | 95% CI[.41,.67] |
|  |  |  |  |  |  |
| *Note.* A significant *b*-weight indicates the semi-partial correlation is also significant. *b* represents unstandardized regression weights. *sr^2^* represents the semi-partial correlation squared. *LL* and *UL* indicate the lower and upper limits of a confidence interval, respectively. * indicates p < .05. ** indicates p < .01. ICV = intracranial volume (mm^3^); CBCL-WD = Child Behavior Checklist Withdrawn-Depressed scale. | | | | | |

| **Supplementary Table 3**  *Moderated regression analysis of grey matter volume for 27 mm^3^ cluster with peak voxel at (-41, 38, 3) based on whole brain analysis.* | | | | | |
| --- | --- | --- | --- | --- | --- |
| Predictor | *b* | *b*  95% CI  [LL, UL] | *sr^2^* | *sr^2^*  95% CI  [LL, UL] | Fit |
| (Intercept) | -0.91** | [-1.39, -0.43] |  |  |  |
| ICV | 0.00** | [0.00, 0.00] | .34 | [.19, .49] |  |
| Sex | 0.14** | [0.08, 0.20] | .11 | [.02, .20] |  |
| Age | 0.02 | [-0.01, 0.05] | .01 | [-.02, .04] |  |
| Maternal Risk | -0.02 | [-0.06, 0.02] | .00 | [-.01, .02] |  |
| CBCL-WD | 0.08** | [0.05, 0.10] | .21 | [.09, .34] |  |
| CBCL-WDxSex | -0.08** | [-0.11, -0.06] | .20 | [.08, .33] |  |
|  |  |  |  |  | *R^2^*  = .593** |
|  |  |  |  |  | 95% CI[.41,.67] |
|  |  |  |  |  |  |
| *Note.* A significant *b*-weight indicates the semi-partial correlation is also significant. *b* represents unstandardized regression weights. *sr^2^* represents the semi-partial correlation squared. *LL* and *UL* indicate the lower and upper limits of a confidence interval, respectively. * indicates p < .05. ** indicates p < .01. ICV = intracranial volume (mm^3^); CBCL-WD = Child Behavior Checklist Withdrawn-Depressed scale. | | | | | |


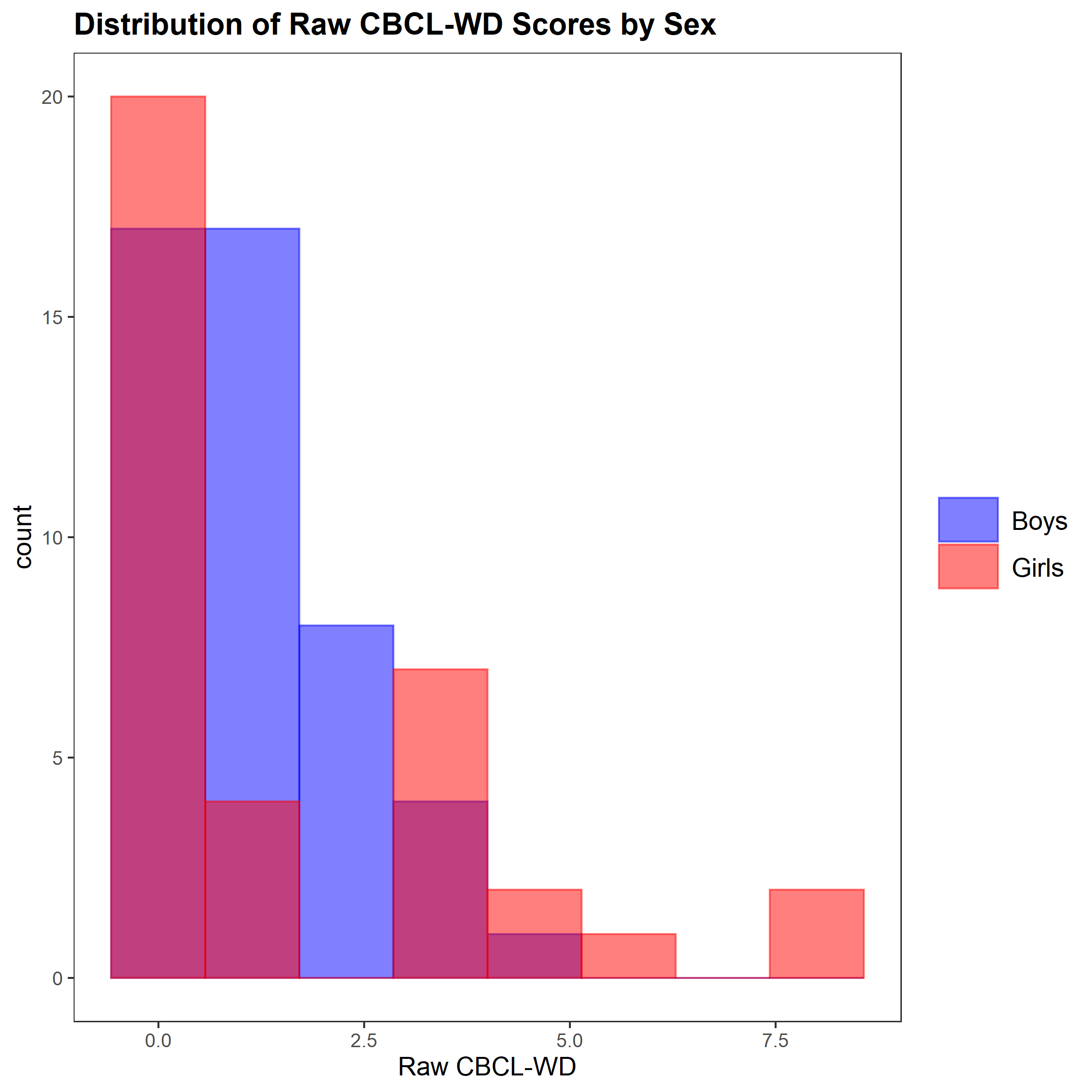


Supplementary Figure 1. Graphical depiction of the distribution of raw Child Behavior Checklist Withdrawn/Depressed (CBCL-WD) subscale scores by sex.


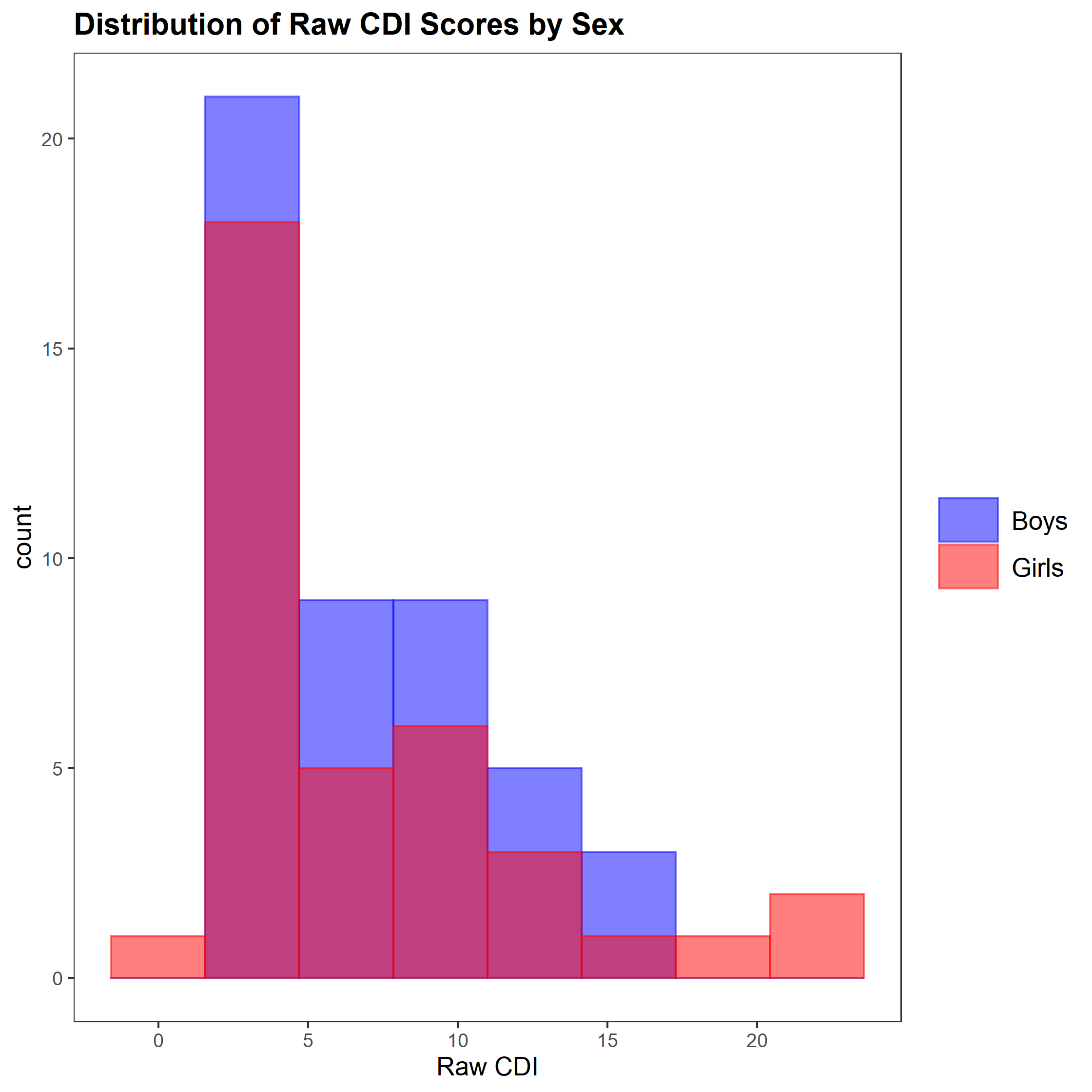


Supplementary Figure 2. Graphical depiction of the distribution of raw Children’s Depression Inventory (CDI) scores by sex.


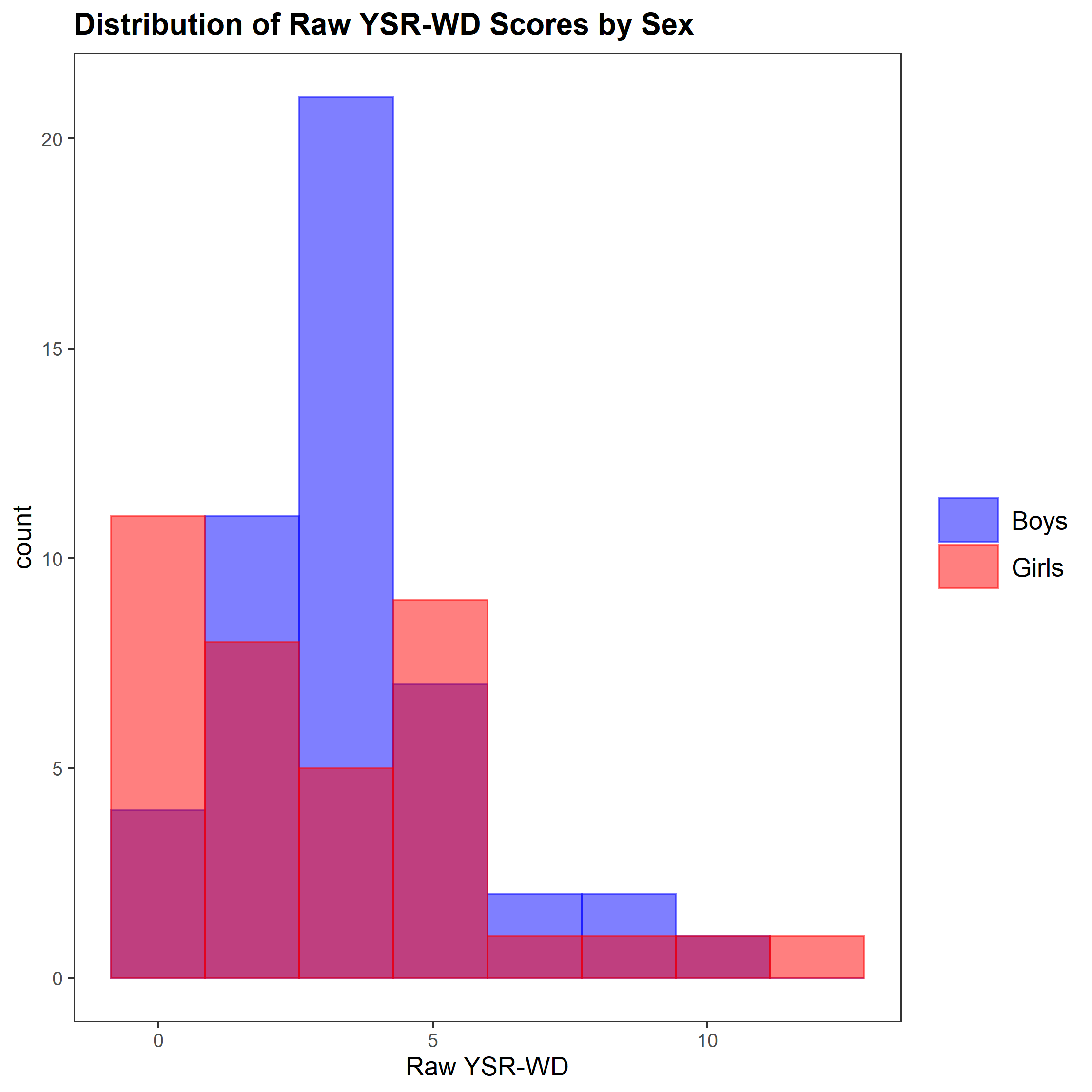
 Supplementary Figure 3. Graphical depiction of the distribution of raw Youth Self-Report Withdrawn/Depressed (YSR-WD) subscale scores by sex.
